# Superhydrophobic Flexible Strain Sensors Constructed Using Nanomaterials: Their Fabrications and Sustainable Applications

**DOI:** 10.3390/nano13192639

**Published:** 2023-09-26

**Authors:** Xiaodong Zhou, Hongxin Zang, Yong Guan, Shuangjian Li, Mingming Liu

**Affiliations:** 1 School of Mechanical Engineering, Shandong University of Technology, Zibo 255000, China; zhouxiaodong784@163.com (X.Z.); zanghongxin2002@163.com (H.Z.); 2 Shandong Inov Polyurethane Co., Ltd., Zibo 255000, China; 3 National Engineering Laboratory of Modern Materials Surface Engineering Technology, Institute of New Materials, Guangdong Academy of Sciences, Guangzhou 510651, China

**Keywords:** flexible sensor, superhydrophobic, conductivity, wetting, nanomaterials

## Abstract

Superhydrophobic flexible strain sensors, which combine superhydrophobic coatings with highly sensitive flexible sensors, significantly enhance sensor performance and expand applications in human motion monitoring. Superhydrophobic coatings provide water repellency, surface self-cleaning, anti-corrosion, and anti-fouling properties for the sensors. Additionally, they enhance equipment durability. At present, many studies on superhydrophobic flexible sensors are still in the early research stage; the wear resistance and stability of sensors are far from reaching the level of industrial application. This paper discusses fundamental theories such as the wetting mechanism, tunneling effect, and percolation theory of superhydrophobic flexible sensors. Additionally, it reviews commonly used construction materials and principles of these sensors. This paper discusses the common preparation methods for superhydrophobic flexible sensors and summarizes the advantages and disadvantages of each method to identify the most suitable approach. Additionally, this paper summarizes the wide-ranging applications of the superhydrophobic flexible sensor in medical health, human motion monitoring, anti-electromagnetic interference, and de-icing/anti-icing, offering insights into these fields.

## 1. Introduction

With the rapid development of technology, the demand for monitoring devices and environments is constantly increasing. Sensors are capable of altering their electrical signals in response to various external stimuli like ion concentration or external pressure. The sensor shows broad applications in production and life, such as industrial production [1], marine exploration [2], environmental protection [3], medical diagnosis [4], biological engineering [5], space exploration [6], smart homes [7], and so on. Traditional sensors typically employ rigid materials like metals or semiconductors as conducting layers, with a deformation measurement range of approximately 5%. These sensors offer advantages such as simplicity, reliability, affordability, and ease of manufacturing. However, their limited flexibility and functionality significantly hinder their future development in the electronic field. As the scope of applications expands, emerging sensors need to possess multifunctionality, including transparency, flexibility, portability, and durability, to adapt to complex service environments.

Flexible strain sensors can meet the above-mentioned requirements. Flexible sensors are designed to withstand bending, twisting, or stretching without sustaining damage. Typically, the design of these sensors incorporates an elastic material, such as metal [8,9], plastic [10], or rubber [11,12], along with a base for securing the measurement elements. The main advantage of flexible sensors is their ability to be customized for different shapes and sizes, making them valuable in applications with limited space or challenging accessibility. The substrate materials used in the flexible sensor commonly include Polyvinyl alcohol [13] (PVA), polyethylene terephthalate [14] (PET), polyethylene naphthalate [15] (PEN), paper [16], textile materials [17], etc. Generally, one or more conductive nanomaterials are used to construct a conductive layer, and the materials used as conductive fillers mainly include carbon-based nanomaterials, metal nanomaterials, nanowire materials, and conductive polymer nanomaterials. At present, carbon-based materials are mostly used as fillers, such as Mxene [18], graphene [19], carbon nanotubes (CNTs), carbon fibers [20], etc. Previous studies have highlighted numerous applications of superhydrophobic flexible sensors in the utilization of nanomaterials and organic polymer materials. However, the continuous advancement of materials science has led to the development of an increasing number of new materials that possess exceptional superhydrophobic properties, serving as a foundation for further research on superhydrophobic flexible sensors. These materials usually consist of metal–organic frameworks (MOFs) [21], conductive polymers [20,22], and so on. At present, flexible strain sensors show broad application prospects in the fields of wearable sensors [23,24], anti-icing surfaces [25,26], anti-electromagnetic interference [27], and motion detection [28,29]. Flexible sensors can be divided into photoelectric sensors [30], photothermal sensors [31], temperature sensors [32], and gas sensors according to different principles. At the same time, flexible sensors can also be designed to have the ability to self-healing [33,34]. This means that if the sensor is damaged, it can restore normal operation by changing its shape or function. Furthermore, researchers have developed flexible sensors with adaptive features that enable automatic adjustments in sensitivity and response time according to the detected stimulus [35,36]. However, a high-humidity environment could cause electrode short circuit, circuit corrosion, or electrode fouling, which further reduce the service life or decrease the service performance. Consequently, the exploration and advancement of superhydrophobic flexible sensors to enhance their adaptability and reliability in such challenging environments have emerged as a crucial area of focus in contemporary scientific and technological investigations.

A superhydrophobic surface is characterized by its minimal affinity for water molecules, effectively preventing water droplets from adhering to the surface and facilitating rolling-off. The superhydrophobic property was initially observed on surfaces of natural living organisms, such as lotus leaves [37,38] and shark skin [39,40]. Wettability, primarily assessed through the contact angle (CA) and rolling angle (SA) of water, is a critical characteristic of solid surfaces. Generally, the contact angle of water droplets on superhydrophobic surfaces is greater than 150°, and the rolling contact angle is less than 10°. There are two main methods to make materials superhydrophobic: one is to form a micro-rough structure on the surface, and the other is to modify the surface with low surface energy. Numerous methods exist for the preparation of superhydrophobic flexible sensors, including the utilization of nanotechnology to fabricate superhydrophobic materials and subsequently integrating them with a flexible substrate to produce such sensors [41]. In addition, there are other methods such as chemical vapor deposition [42,43], electrochemical deposition [44], and sol–gel methods [45,46] to prepare superhydrophobic materials, which can then be combined with a flexible substrate to produce superhydrophobic flexible sensors. This unique physical property means that superhydrophobic materials have broad application prospects in many fields, including surface self-cleaning [47,48,49,50], oil–water separation [51,52], anti-corrosion [53,54,55], anti-icing [56,57], and other aspects. However, the use of superhydrophobic materials in sensor fabrication often presents challenges such as limited flexibility, difficult integration, and high cost. In order to address these issues, researchers have started investigating the integration of superhydrophobic materials with flexible electronic technology to create superhydrophobic flexible sensors that possess de-icing capabilities [58], self-healing properties [59,60] or flame retardant properties [61]. Such sensors not only exhibit adaptability to extreme environmental conditions, but also possess excellent flexibility and integration, making them easily applicable to a wide range of devices. Currently, research on superhydrophobic flexible sensors is in a highly active stage of development, with the corresponding research findings yet to transition from laboratory settings to industrial production.

In this paper, the chemical composition, geometric morphology, and surface wettability of the surface microstructure are discussed. Based on the limitation of the superhydrophobic theoretical model, the effects of the existing Wenzel model and Cassie model and the structure of the nano-layer on the superhydrophobic properties are analyzed. The sensing mechanism, which is based on the tunneling effect and percolation theory, is thoroughly examined. This paper introduces the fundamental performance parameters of superhydrophobic flexible strain sensors, encompassing sensitivity, response time, sensing range, and cycle stability. Various processing methods including photolithography, chemical etching, dip coating, and electrodeposition are systematically discussed. Finally, the applications of superhydrophobic flexible sensors in human health detection [62], human motion measurement [63], anti-icing, and de-icing are summarized. This review will provide a reference for designing new superhydrophobic flexible sensor methods and realizing batch production.

## 2. Superhydrophobic Flexible Sensor Mechanism

### 2.1. Overview of Superhydrophobic Surfaces

Since the 1960s, with the development of scanning electron microscopy, scientists have published a large number of articles about the cutin structure of plant surfaces, which has a great influence on surface properties [64]. The hydrophilicity of red rose petals [65], the transition from hydrophobicity to hydrophilicity of rape leaves [66], the hydrophobicity of shark skin [67], the ability of water strider legs to stand on the surface of water, and other superhydrophobic properties are all related to surface wettability.

**Figure 1:**
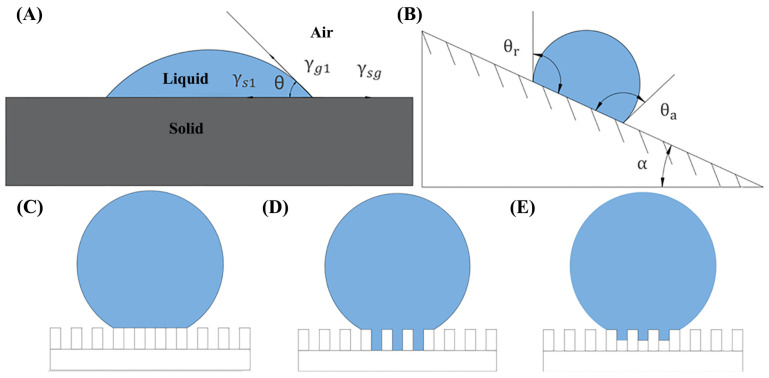
(**A**) Contact angle of droplet on a solid surface. (**B**) Rolling and contact angle of dropleton an inclined surface. (**C**–**E**) Cassie–Baxter theory model, Wenzel theory model, and transient Wenzel–Cassie model.

Surface wetting is a common phenomenon in everyday life, referring to the ability or tendency of a liquid to spread on a solid surface when in contact, replacing the gas through surface tension. In order to evaluate the wettability of a solid surface, two parameters are introduced, namely the static contact angle (CA) and the rolling angle (SA). The static contact angle is defined as the angle between the liquid–solid interface line and the liquid–gas interface tangent line at the intersection point of the gas–liquid–solid three-phase interface when the liquid is at rest and in equilibrium with the solid surface (Figure 1A,B). The superhydrophobic surface is defined as the angle that satisfies CA ≥ 150°. The adhesion of droplets on a superhydrophobic surface varies, with droplets that have low adhesion being more likely to roll off an inclined surface. To quantify this behavior, the concept of a rolling angle is introduced. The rolling angle is defined as the critical angle between the inclined surface and the horizontal plane at which the droplet begins to roll on the inclined surface. The superhydrophobic surface usually requires SA < 10°. For a liquid drop on an ideal solid surface, the magnitude of the contact angle is given by Young’s equation:

$$\cos\theta =\frac{\gamma_{\mathrm{sg}}-\gamma_{\mathrm{sl}}}{\gamma_{\mathrm{gl}}}$$

where $\gamma_{\mathrm{sg}}$, $\gamma_{\mathrm{sl}}$, $\gamma_{\mathrm{sl}}$ are the interfacial tensions between solid and gas, solid and liquid, and gas and liquid, respectively. The equation gives a direct judgment of hydrophilicity or hydrophobicity on the surface of solid materials from the mathematical level. The surface is hydrophobic when the angle is greater than 90° and hydrophilic when the angle is otherwise [68].

The premise of using Young’s equation is that the solid surface is uniform, but it is difficult for real solid materials to satisfy this condition in terms of composition and morphology. Consequently, scientists have proposed various models to describe water droplet contact on rough surfaces. (1) The Cassie model (Figure 1C) suggests that a droplet on a composite surface of liquid and air cannot fill the grooves and instead forms compound contact. (2) The Wenzel model [69] suggests (Figure 1D) that when a water droplet is on a rough solid substrate, it can infiltrate and fill the grooves on the rough structure, resulting in wet contact. The relationship between the actual contact angle $\theta_{W}$ of a rough surface according to the Wenzel model and the contact angle θ in Young’s equation is

$$\cos\theta_{W}=\frac{r(\gamma_{\mathrm{sg}}-\gamma_{\mathrm{sl}})}{\gamma_{\mathrm{gl}}}=r\cos\theta$$

where *r* is defined as the surface roughness factor, whose numerical value is equal to the ratio of the actual surface area to the projected area. When *r* > 1, increasing the roughness of the solid surface makes the hydrophobic surface more hydrophobic and the hydrophilic surface more hydrophilic.

Scientists Cassie and Baxter studied and proposed the Cassie–Baxter theoretical model [70] (Figure 1E). When a droplet is located on a rough surface, the liquid does not completely enter the rough structure but is in a composite contact form of solid, liquid, and gas. Because the droplet is usually larger than the size of the rough structure, the droplet and the rough surface will have air entrapment, and the formed air protective layer will further prevent the droplet from wetting. Therefore, the Cassie–Baxter theoretical model formula is proposed:

$$\cos\theta_{c}=\left(1-f\right)\cos180^{{}^{\circ}}f\cos\theta =f\left(\cos\theta +1\right)\;-\;1$$

$\theta_{c}$ denotes the contact angle at the time of composite contact and f is the area ratio of the contact interface between the liquid and the solid in the rough surface covered by the liquid. From the formula, the smaller the proportion of the solid–liquid interface, the better the hydrophobicity.

The contact angle is only a criterion of static wettability of the superhydrophobic surface. The roll angle can be used as a measure of the dynamic wetting process, which is the angle of inclination at which a small droplet begins to roll on an inclined surface. Only when the solid surface inclines to the rolling angle (α) and the front and rear slopes of the droplet reach the advancing angle $\theta_{a}$ and the receding angle $\theta_{r}$ does the liquid begins to roll. The rolling of the droplet is caused by the hysteresis of the actual contact angle of the solid surface. Nasonovsky and Bhushan derived the formula for the rolling angle of a drop on a rough surface from Cassie’s equation:

$$\cos\theta_{n}-\cos\theta_{r}=R_{f}\int \mathrm{sl}\left(\cos\theta_{a0}-\cos\theta_{r0}\right)+H_{r}$$

where the first term on the right is the intrinsic adhesion hysteresis of a smooth surface and $H_{r}$ is surface roughness effects.

The above two models are extreme cases, and the actual wettability of solid surfaces is somewhere between them. This phenomenon is called the Wenzel–Cassie transition state model. Bico and Marmur jointly proposed the apparent contact angle threshold $\theta_{T}$:

$$\cos\theta_{T}=(f_{1}-1)(r\;-f_{1})$$

$$\cos\theta_{M}=r_{1}f_{1}\cos\theta_{1}+f_{1}-1$$

where $\theta_{M}$ is the apparent contact angle of the transition state, $r_{1}$ is the roughness of the droplet and the solid surface, $f_{1}$ is the integral number of the phase surface contacting the droplet, and $\theta_{1}$ is the intrinsic contact angle contacting the droplet.

At present, superhydrophobic surfaces have shown great potential in the fields of waterproofing, corrosion resistance, and anti-icing and have been widely used in transportation, aerospace, and building materials.

### 2.2. The Conductive Mechanism of Sensors

The main focus of this study is on the sensing principle of superhydrophobic flexible sensors, including the seepage theory and tunneling effect. Then, the preparation of superhydrophobic surfaces is discussed, with a particular emphasis on the wetting mechanism. Superhydrophobic flexible sensors are still in development and have not reached the point of industrial production. In this study, the sensor preparation process was chosen to avoid the conventional approach commonly used by most researchers, focusing on surface wetting properties. This deviation is attributed to the misalignment of current work processes with modern principles of environmental protection and conservation. Future research in this field will primarily concentrate on theoretical analysis and investigation of the sensors’ operating mechanism from multiple perspectives.

#### 2.2.1. Percolation Theory of Composite Materials

The theory of osmosis was first proposed by the British mathematician Hammersley in his study of the flow of fluids in disordered porous media. With the change in the mass fraction of conductive fillers in conductive polymer composites, the resistance ratio of the materials also changes. In the experiment, the resistance ratio of the composites changed little with the concentration of conductive filler in a certain range. Upon reaching a critical point, an increase in the content of conductive filler led to a significant decrease in the resistance ratio of the composites. As the content of conductive filler increases, the resistance ratio of the composites approaches a constant value, indicating that changes in the concentration of conductive filler have no significant impact on resistivity. This is called the percolation phenomenon, which can be explained by the theory of conductive pathways [71].

At low concentrations of conductive fillers, they are dispersed within the polymer matrix with limited contact, resulting in high resistivity of the composite. Because of the small concentration of conductive filler, it fails to form a conductive network and exhibits insulating properties. When the conductive filler reaches a certain concentration, the resistivity decreases rapidly, and this concentration is called the percolation threshold. With the further increase in the conductive filler concentration, many complete conductive paths are formed in the composites, and the resistivity of the composites tends to a constant value. The increase in the conductive filler concentration has no obvious effect on the resistivity. The theory of conductive paths explains the formation of the conductive path in conductive composites and the relationship between the conductivity of composites and the concentration of conductive fillers, but it also has some limitations. The theory of conductive paths is only based on the assumption of external experimental characteristics. It suggests that when conductive fillers come into contact or are closely spaced, a conductive path can be formed, allowing the generation of current. However, it remains a theoretical model. Due to van der Waals forces, most of the conductive fillers exist in the form of aggregates.

#### 2.2.2. Tunneling Effect of Composite Materials

At low volume fractions of conductive nanomaterials, adjacent conductive nanomaterials are separated by insulating polymer materials. However, when the distance between them is within the cut-off distance, electrons can still transfer between the nanomaterials, resulting in conductivity of the composite, referred to as the tunneling effect [72]. Tunneling effect theory uses quantum mechanics to study the relationship between the resistivity of materials and the gap of conductive particles. The gap between conducting particles is directly influenced by the concentration of conducting particles within the material and the surrounding temperature. According to tunneling theory, composites conduct electricity because of the migration of electrons between conductive particles during vibration. If the spacing between two adjacent conductive particles falls within the cut-off distance, the tunneling resistance can be determined using the Simmons tunneling theory:

$$R=\frac{V}{\mathrm{AJ}}=\frac{h^{2}d}{Ae^{2}\surd 2m\lambda }\exp(\frac{4\pi d}{h}\sqrt{2m\lambda })$$

wherein A represents the tunnel junction cross-sectional area, *J* represents the tunneling current density, *V* represents the potential difference, *e* represents the charge quantity, *m* represents the electron mass, *λ* represents the polymer energy barrier, *h* represents the Planck constant, and *d* represents the distance between adjacent nanomaterials. According to the formula, when the distance d between adjacent nanomaterials increases, the tunneling resistance increases, and when the strain is released, the distance d between adjacent nanomaterials decreases continuously, so the conductive path is restored.

Both carbon nanotubes and graphene nanosheets are elastic nanomaterials with reversible structure and morphology. In conductive polymer composite materials, carbon nanotubes or graphene nanosheets are utilized as conductive fillers. These fillers are then wound and folded within a flexible substrate to create a conductive network. Upon applying strain to a conductive polymer composite, the elastic nano-conductive fillers undergo a transformation from wrapped or folded states to extended states. This transformation leads to increased isolation distances and changes in resistance. Once the strain is released, the conductive network returns to its original state due to the restorative properties of elasticity. This behavior allows for a resistance response under flexible strain.

## 3. Performance Parameters of Superhydrophobic Flexible Sensors

The performance of the superhydrophobic flexible sensor is evaluated based on its sensitivity, response time, sensing range, and cycle stability. Additionally, the sensor is expected to possess high tensile strength, durability, corrosion resistance, and self-cleaning capabilities, enabling it to meet the diverse requirements of various application scenarios.

### 3.1. Research on Sensor Sensitivity

Sensitivity is the basic characteristic of the sensor, which refers to the relationship between the output and input of the sensor, and is determined by the sensitivity coefficient (gauge factor, GF), with the following formula:

$$GF=\frac{\left(∆R/R_{0}\right)}{\varepsilon }$$

where Δ*R* represents the relative change in resistance and *ε* represents the strain coefficient. High sensitivity has a great impact on the detection quality of superhydrophobic flexible sensors. At present, many sensors can only maintain stable sensing performance in a limited area, so high sensitivity and high stretching ability are the research focus of researchers.

**Figure 2:**
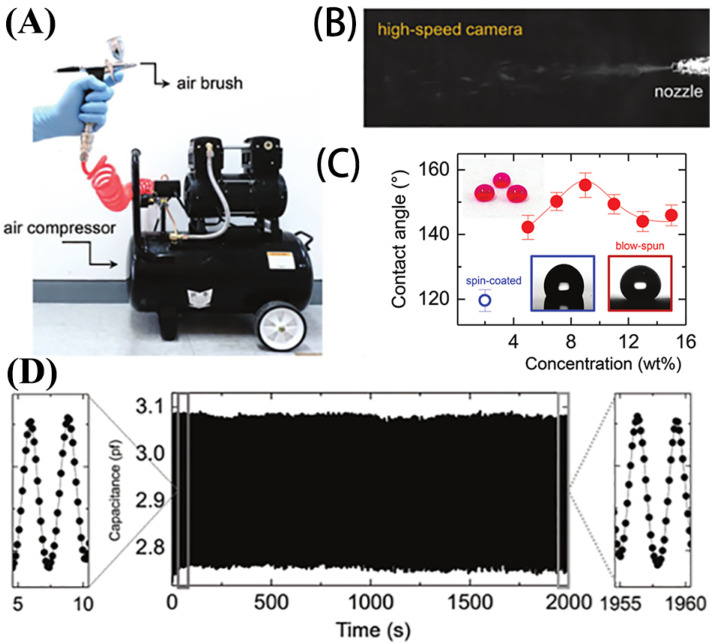
(**A**) Spray gun and air compressor units as part of jet spinning equipment. (**B**) High-speed cameras were used to capture photos of the nanofibers being ejected from the spray gun nozzle. (**C**) Contact angle and water dripping on the fabric for different solution concentrations of the preparation of PVdf HFP fabric. (**D**) The strain sensors underwent over 1000 stability tests, and the durability tests revealed consistent values in the time magnification plots at the beginning (5–10 s) and end (1955–1960 s).

Jia et al. [73] dispersed carbon nanotubes in vulcanized silicone rubber (RTV) to form a conductive network and prepared a super-stretched MWCNT/RTV composite sensor. Additionally, the sensor’s surface, formed using the carton as a mold, exhibits super-hydrophobic properties. The sensor has high sensitivity (GF of 2.1 to 214), a wide sensing range (up to 447% strain), low resolution (<0.2% strain), dynamic durability (stretching more than 10,000 times at 50% strain), and superior superhydrophobicity to most reported waterproof sensors. Chu et al. [74] developed a superhydrophobic strain sensor with a gradient structure. This was achieved through a straightforward one-step pre-stretching method, which biaxially stretched a VHB substrate film, transferred a reduced graphene oxide (rGO) film onto its surface, and coated it with a PDMS solution for a superhydrophobic surface. The strain sensor has high sensitivity (maximum measurement coefficient 1199.10), a wide strain range (up to 400%), strong stability (3000 cycles), a low detection limit (0.1% strain), and fast response (88 ms). It can be used to monitor the full range of human motion. Ho et al. [75] fabricated a simple and cost-effective non-woven fabric using blow-spinning technology. They blow-spun Poly(vinylidene fluoride-co-hexafluoropropylene) (PVdF-HFP) dissolved in acetone onto the target substrate and vertically stacked the PVdF-HFP dielectric fabric with SWCNT/PVdF-HFP conductive fabric to make an all-fabric capacitive strain sensor (Figure 2A,B). The sensor has a drop contact angle of up to 158°, strain sensitivity of up to 134, and a minimum detection strain of 0.03% (Figure 2C).

### 3.2. Reduction in Sensor Response Time

The response time is one of the important parameters that affect the performance of superhydrophobic flexible sensors and has a great influence on user experience. This parameter is the time that the sensor takes to achieve steady state. The smaller the response time, the faster the sensor responds to sudden strain, which is of great significance for the accurate measurement of physiological signals such as pulse.

Wu et al. [76] synthesized a conductive and superhydrophobic aerogel for piezoresistive pressure sensors. The aerogel was composed of 1H,1H,2H,2H-perfluorooctyltriethoxysilane (FAS)-modified reduced graphene oxide, carbon nanotubes, and chitosan (F-rGO@CNTs/CS). The sensor can be made superhydrophobic due to the rough structure composed of carbon nanotubes and pores as well as the low surface energy of FAS. And the contact angle is up to 154°. At the same time, due to the porous structure of the aerogel and the synergistic effect of the carbon nanotubes and reduced graphene oxide, the aerogel sensor achieves high sensitivity and fast response (time < 170 ms). Based on a biomimetic strategy inspired by the lotus leaf and scorpion, Liu et al. [41] achieved superhydrophobic characteristics and ultra-sensitive vibration sensing ability, respectively. A paper-based strain sensor with high sensitivity and water repellency has been successfully prepared by adding MWCNTs and SiO_2_ nanoparticles into HDMS and n-butyl acetate and coating with PDMS. The strain sensor exhibits a strain coefficient of 263.34, excellent stability over 12,000 cycles, a water contact angle of 164°, and a rapid response time of 78 ms, surpassing that of other sensors.

### 3.3. Research on Increasing the Sensor Sensing Operating Range

Since the superhydrophobic flexible sensor is often used as a wearable device, wearer comfort and working stability are the basic requirements. Furthermore, considering that the skin of the feet and joints can stretch and contract at a rate of approximately 55%, the sensor must also possess a wide sensing working range to cater to daily applications.

Wang et al. [77] prepared a flexible conductive polymer foam composite (CPFC) with a lotus leaf excitation microstructure. They fabricated hemispherical arrays on the skeleton of the polymer foam, and these arrays were decorated with CNTs to form a conductive network. The CPFC superhydrophobic sensor fabricated showed excellent corrosion resistance. It has good hydrophobicity and the contact angle is about 155 degrees. The sensor demonstrates stable conductivity, excellent sensing stability, and durability (over 2300 cycles), allowing for a consistent sensing signal within the temperature range of 20 °C to 80 °C. Additionally, it exhibits extremely high compressibility and a wide working range, enabling the detection of strains ranging from as small as 5% to as large as 180%. Yao et al. [78] utilized surface penetration technology to create a gradient conductive network by introducing carbon black (CB) from the surface of natural rubber latex (NRL) gloves to the interior of the gloves. The CB formed a rough micro–nano-scale structure and constructed a superhydrophobic surface. The gradient conductive network sensor has a wide strain detection range (0.05~300%) and good stability. Flexible sensors are capable of detecting a broad range of human movements, ranging from subtle changes like wrist pulse to more extensive motions like joint movement. The method of mounting a flexible sensor on a flexible substrate can realize a wide pressure detection range (1.7~2900 kPa).

### 3.4. Research on Sensor Cyclic Stability

Cycling stability refers to the fact that the flexible sensor still has intact sensing and mechanical properties after multiple stretch–release cycles when subjected to a certain amount of pressure. With multiple cycles, the conductive particles on the surface or inside are broken, the resistance suddenly increases, and distortion occurs, thus reflecting the durability of the sensor to a certain extent.

Choi et al. [79] coated silver nanoparticle (Ag NP)–polymer (SBS) composites on Kevlar fibers and then treated the prepared fibers with a self-assembled monolayer (SAM)-forming agent to obtain waterproof and self-cleaning properties on the surface. The contact angle of the 1H,1H,2H,2H-perfluorohexanethiol (PFDT)-treated conductive fiber is 150° and the root mean square (RMS) roughness is 1.171 μm. The conductive fiber exhibits a conductivity of 0.11 Ω/cm and possesses excellent mechanical durability. When folded 10,000 times at 150°, the electrical properties of the conductive fiber show no significant change, with a slight increase in resistance from 0.11 Ω/cm to 0.15 Ω/cm. Liu et al. [80] fabricated a composite strain sensor using SR/MWCNTs/laser-induced graphene (LIG)/SR using laser direct writing technology and prepared a highly stable crosslinked conductive network layer and a high-performance superhydrophobic layer (CA is 155°) with excellent anti-icing (36 min at −5 °C) and photothermal deicing (88 s@1 W/cm^2^@NIL) performance. The sensor has a high measurement factor of 667, a wide strain detection range spanning from 0% to 230%, and a stable sensing response for over 2500 cycles at 200% strain (Figure 2D). Additionally, it can recover to its initial value with regular changes.

## 4. Surface Preparation Technology of Superhydrophobic Flexible Sensors

The wettability of a solid rough surface primarily depends on two factors: surface roughness and surface energy. Increasing surface roughness reduces the contact area between the liquid and the surface, thereby decreasing the force between them. Additionally, reducing the surface energy of solids allows for liquid adhesion to the surface. Consequently, there are two methods for preparing superhydrophobic surfaces: endowing a rough surface with low surface energy or texturing a rough structure for a material’s surface with low surface energy. The reported preparation methods of superhydrophobic surfaces are generally divided into physical methods and chemical methods. This paper provides an overview of various methods such as photolithography, chemical etching, dip coating, electrodeposition, and more. According to different application sites, the corresponding processing methods are selected. The following methods are briefly introduced.

### 4.1. Photolithography Manufacturing of Superhydrophobic Flexible Sensors

Upon ablation using a laser engraving machine, the surface of a composite material becomes significantly rough, forming a regular convex structure. Highly stable superhydrophobic surfaces are prepared by adjusting the laser scanning interval, scanning speed, and power. This approach eliminates the need for cumbersome chemical modification and mitigates issues such as weak interface strength between the coating and the substrate, low adhesion, and easy peeling of the coating.

**Figure 3:**
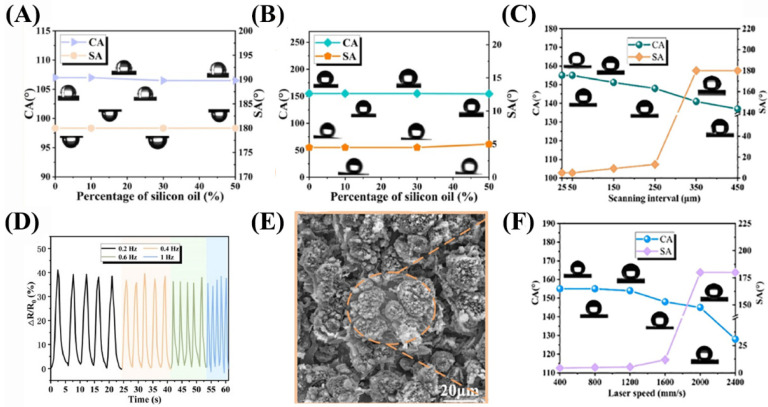
(**A**,**B**) Before and after laser treatment. Adding different contents of silicone oil will not affect hydrophobic properties and does not penetrate the PDMS crosslinking network. (**C**) The influence of laser scanning interval on the CA and SA. (**D**) The sensor under different strain frequencies. (**E**) Surface electron microscopy images of after laser ablation. (**F**) The influence of scanning speed on the CA and SA.

Chen et al. [81] employed the laser-induced graphene (LIG) process to fabricate a flexible droplet generator (DEG) electrode with structural and hydrophobic properties on the surface of fluorinated ethylene propylene (FEP)-coated polyimide (PI). It has high power conversion efficiency and can generate a peak power density of 47.5 W m^−2^ from the impact of 105 µL water droplets at a 25 cm height. The fabricated sensor surface has a large advancing angle (157.4°), a large receding angle (152.9°), and a small hysteresis angle (0.8°), with a surface sheet resistance of 120 Ω square^−1^. When the temperature increases from 20 °C to 45 °C, the output voltage and current remain above 87% of the original value, and the output voltage and current of the device decrease by 30% after 10,000 times of power generation and decrease by 50% after 30,000 times of power generation. Liu et al. [82] uniformly dispersed carbon nanotubes (CNTs) in a polydimethylsiloxane (PDMS) matrix to construct a stable conductive network. The PDMS/CNT composites irradiated with a laser showed excellent superhydrophobicity, with a contact angle of 155° and a sliding angle of 4.5° (Figure 3A–C). Good sensitivity with a measurement coefficient of 3.1 and a wide sensing range up to 100% was achieved (Figure 3D). Upon laser ablation, the surface of the PDMS/CNT composites became rough and formed regular convex structures (Figure 3E). The influence of scanning speed on the their wettability is shown in Figure 3F. Under stretching conditions, the PDMS/CNT composites maintained good superhydrophobic performance, with a surface contact angle of 152 degrees and a rolling angle of 8.5 degrees when stretched by 200 percent.

### 4.2. Surface Etching Manufacturing of Superhydrophobic Flexible Sensors

Utilizing chemical reagents to corrode the surface of materials and create a micro-rough structure is a convenient and expeditious method for achieving a superhydrophobic interface. Physical etching primarily employs plasma ablation, a cost-effective and environmentally friendly technique that is straightforward to execute. The principle involves the selective bombardment of sample surfaces by electrons under the influence of voltage, enabling controlled etching through the regulation of electron irradiation intensity and duration.

**Figure 4:**
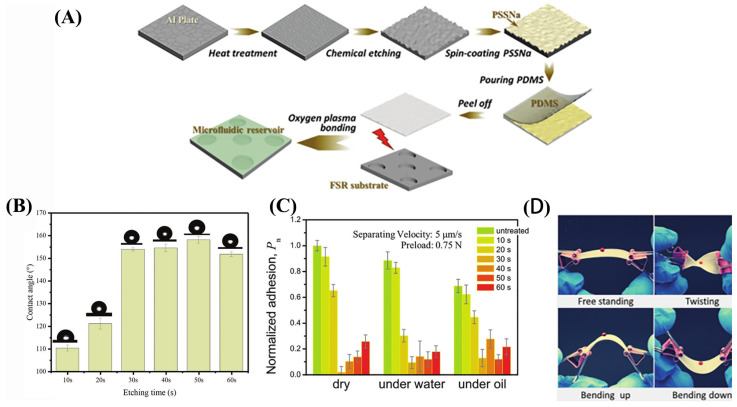
(**A**) Schematic PDMS replicas of the manufacturing process. (**B**) Different etching time template copying of PDMS surface water contact angle. (**C**) Normalized adhesion (0–60 s) of PDMS in air, water, and oil for different etching templates. (**D**) Freedom, distortion, and upward and downward bending loss of droplets on the PDMS replicas under the condition of the optical image.

Cheng et al. [83] employed chemical etching and soft lithography techniques to etch superhydrophobic structures onto aluminum plates. They then utilized polydimethylsiloxane (PDMS) to replicate the microstructure of the aluminum plates, resulting in low-adhesion surfaces suitable for epidermal microfluidic devices (Figure 4A). In comparison to soft layers prepared using previous methods, the PDMS layer exhibits significantly reduced adhesion. As shown in Figure 4B, the adhesion properties of several PDMS replicas at different etching times were studied. The top-performing replica exhibits a contact angle exceeding 150° and a rolling angle below 5° (with a contact angle hysteresis of less than 15°). Notably, as shown in Figure 4C,D, this layer demonstrates the ability to sustain ultra-low adhesion with various engineering materials under different environmental conditions, including dry, underwater, and oil environments. Moreover, it exhibits excellent durability (over 1 month) and stability (at least 20 cycles) of use.

### 4.3. Dip Coating Manufacturing of Superhydrophobic Flexible Sensors

Dip coating is an industrial coating process used for the mass production of products and is also used in many chemical and nanomaterials research projects to produce coatings. Dip coating can be conducted as a continuous rolling process for flexible substrates, such as fabrics, or as a simple dipping and removal process for coating 3D objects. The final product comprises a substrate and a coating, with a coating or film consisting only of the dried or cured coating. The dipping time, pulling speed, dipping cycle times, solution composition, solution concentration, and temperature have an influence on the final outcome of the dipping process.

**Figure 5:**
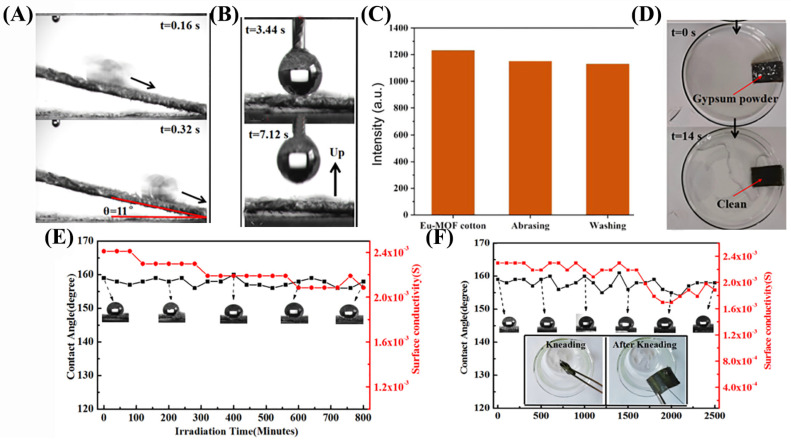
(**A**) Sliding water droplets at a certain angle. (**B**) Water drops suspended on the coating. (**C**) Eu-MOFs coating cotton cloth after rubbing and washing of luminous intensity. (**D**) Gypsum powder on the surface, water spraying, the surface without a trace. (**E**) Light influence on the layer conductivity and hydrophobic. (**F**) Variation in hydrophobicity and surface conductivity of flexible fabric coatings with kneading cycle and hydrophobicity.

Xiao et al. [21] fabricated micro–nano europium metal–organic frameworks (Eu-MOFs) by introducing a blocking agent into a luminescent lanthanide organic framework and then coating them onto cotton fabrics via hot pressing. As shown in Figure 5A,B, the copper ion sensor fabricated using this method had excellent hydrophobicity. The selective sensing ability of the sensor for Cu^2+^, K^+^, Zn^+^, and other metal ions was verified using emission spectra (Figure 5C). With the increase in Cu^2+^ concentration, the fluorescence of the fabric decreased obviously and almost disappeared in 0.1 mol/L Cu^2+^ solution. In the range of 10^−6^–10^−2^ mol/L, I and I_0_/I at 618 nm changed exponentially with Cu^2+^ concentration. Figure 5D–F depicts the alterations in superhydrophobicity and sensing performance of the cotton fabric, along with the changes in sensing performance of the Eu-MOFs coating. Pei et al. [84] prepared Ag-Zn(OH)_2_@STA composite coatings on flexible fabric substrates by impregnating silver nitrate (AgNO_3_·6H_2_O) into a precursor solution containing stearic acid (STA). The composite coating comprises a network structure composed of Zn(OH)2@STA nanosheets and numerous small Ag@STA nanoparticles. This unique coating structure imparts superhydrophobicity and conductivity to the fabric, resulting in a contact angle exceeding 150 degrees. The conductivity is almost linear with the bending angle theta, and the coating is very suitable for angle sensing; its mechanical durability is good, and the electrical conductivity changes little after 900 kneading test cycles: from 2.29 × 10^−3^ S to 1.98 × 10^−3^ S, a decrease of only 13.5%. The main reason for the decline in performance is the continuous shedding of silver nanoparticles during kneading.

### 4.4. Electrodeposition Manufacturing of Superhydrophobic Flexible Sensors

Electrochemical deposition is a chemical method used to achieve superhydrophobic modification by reducing metal cations on material surfaces, which leads to the formation of rough structures. This method is not only simple and easy to control but also allows for precise regulation of coating thickness and morphology. It is a crucial technology for large-scale preparation of superhydrophobic surfaces and has found applications in the production of photovoltaic and solar modules.

**Figure 6:**
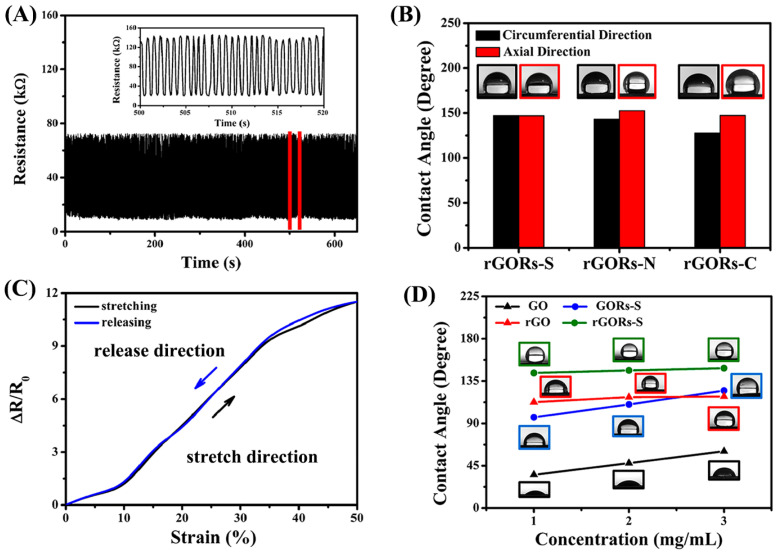
(**A**) Change in resistance for 1000 cycles with constant stress. (**B**) rGORs, rGORsS-N, and rGORs-C along the axial and circumferential contact angle. (**C**) The 0–50% strain range of the tensile/release hysteresis loop. (**D**) GORs is rGORs-S-S and rGOR contact angle under different concentrations of GO.

Niu et al. [42] used bio-activated polydopamine to strengthen the interface between AgNPs and fabric, and modified the surface of the AgNPs using the fluorine-containing agent 1H,1H, 2H, 2H-perfluorodecanethiol (PFDT). The surface prepared exhibits superhydrophobic properties with a contact angle of 152° due to the high surface roughness of AgNPs and the low surface energy of PFDT. Meanwhile, it has good corrosion resistance to water, acid/alkali solution, milk, coffee, etc. The electronic textile has a low sheet resistance of 0.26 ohm/sq and a high conductivity of 233.4 S/cm. Moreover, it demonstrates exceptional mechanical properties, including a tensile strength of 0.06 MPa, Young’s modulus of 0.34 MPa, and an elongation at break of 27%. Song et al. [85] conducted simulations on different sections of a curved substrate to produce layered buckling patterns of reduced graphene oxide (rGO). By compressing two cloths on the cylindrical substrate, the resulting reduced graphene oxide ridges (rGORs) exhibited a significantly increased level of hierarchy and directionality, along with high axial sensitivity (Figure 6A,B) and excellent chemical protection performance. The oriented rGORs are superhydrophobic and strain-sensitive (Figure 6C), with a contact angle greater than 150°(Figure 6D), high sensitivity (maximum measurement factor up to 48), high uniaxial stretchability (300–530%), and ultra-high area stretchability (up to 2690%).

In addition to the above-mentioned methods, the mold method [86], electrostatic spinning [87], spraying method [88] and other methods are also used to prepare superhydrophobic flexible sensors. The selection of suitable processing methods depends on the equipment requirements and environmental conditions, as each method has its own strengths and limitations. Exploring the combination of multiple methods for sensor fabrication is a promising research direction. At present, the preparation process of most superhydrophobic flexible sensors is complex, and transitioning them from the laboratory to practical applications is challenging. The quest for a simplified preparation process is a significant challenge. Consequently, numerous researchers have embarked on investigating straightforward methods for constructing unique superhydrophobic surfaces.

### 4.5. Electrospinning

Electrospinning is an important method to prepare nanofibers and is a convenient, simple, and pollution-free technology. The principle is that the charged fluid flows and deforms in the electrostatic field, and the fibrous material is finally obtained from the movement of the programmable collector and the nozzle. Electrospinning, as an efficient additive manufacturing technology, is easy to use to generate complex patterns, with high repeatability and low cost, and is increasingly popular among researchers.

**Figure 7:**
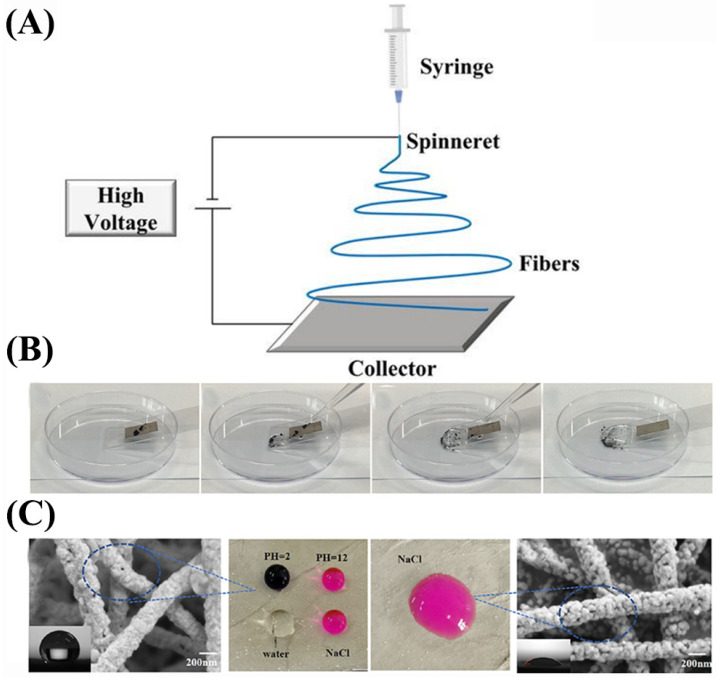
(**A**) Electrostatic spinning equipment. (**B**) Experiment proving that the sample has good self-cleaning ability. (**C**) Electron microscope images of samples and different pH wettability of the surface of the liquid in the sample.

Lin et al. [89] utilized acid-modified carbon nanotubes (ACNTs) to decorate the surface of electrospun thermoplastic polyurethane (TPU) nanofibers. Additionally, they incorporated silver nanowires (AgNWs) into the gaps of the fiber membrane to establish a conductive network. A protective structure of PDMS was introduced, resulting in the fabrication of a WCNC (TPU/ACNTs/AgNWs/PDMS) composite. The composite exhibits superhydrophobicity, resistance to harsh environments, and self-cleaning properties (contact angle can reach 154 °C, and it remains at 150 °C during a two-hour acid resistance experiment). It also demonstrates high conductivity (up to 3506.8 S/m), sensitivity, GF up to 1.36 × 10^5^, and a wide working strain range of 38% to 100%. Furthermore, it maintains excellent stability even after undergoing multiple stretch release processes (up to 1200 times, 70% strain). Drawing inspiration from the hierarchical structure of nacre, Li et al. [90] fabricated lightweight, flexible, and superhydrophobic PAN@SiO_2_-Ag composite nanofiber membranes. (Figure 7A) They achieved this by incorporating SiO_2_ into a solution of N,N-dimethylformamide (DMF) and introducing polyacrylonitrile (PAN) powder using needle-based electrospinning components. It has high electromagnetic interference shielding performance, with an average shielding efficiency (SE), specific shielding efficiency (SSE), and absolute shielding effectiveness SSE/t of 82 dB, 367 dB cm^3^g^−1^ and 73,478 dB cm^2^g^−1^, respectively. The composite demonstrates strong hydrophobicity (Figure 7B), as evidenced by a water contact angle of 156.99 degrees. It also exhibits excellent conductivity in harsh environments, allowing solutions with varying pH values to roll on the surface (Figure 7C) while maintaining a conductivity level above 160 S/m.

## 5. Application Direction of Superhydrophobic Flexible Sensors

Superhydrophobic flexible sensors find extensive applications in various fields such as human health detection, motion detection, anti-electromagnetic interference, and anti-icing/de-icing. However, each application field has distinct sensor requirements. In the context of human health detection, high requirements are placed on wearer comfort, sensitivity, and response time. On the other hand, human motion detection requires the sensor to possess a wide strain range and cycle stability. De-icing/anti-icing requires the sensor to have high superhydrophobic performance. Depending on the application scenario, researchers must carefully choose suitable sensing materials and preparation technologies to fabricate distinct conductive and superhydrophobic structures.

### 5.1. Flexible Sensors for Human Health Detection

Wearable bioelectronic monitoring equipment can provide valuable human health information. Intelligent flexible sensors enable real-time tracking of physiological signal data, including pulse electrocardiograms and electroencephalograms, without the need for complex detection programs. After receiving feedback from the receiver, medical staff can process and treat in time, which is an important part of telemedicine systems. Furthermore, the utilization of flexible sensors in human health detection aligns with the public’s aspirations for healthier lifestyles and medical convenience [91]. For the technology of flexible sensors for physiological signal detection, it is the premise to obtain stable and high-quality physiological signals to ensure stability, durability, comfort, and ventilation. Currently, there is a wealth of research results, predominantly centered on developing sensors with enhanced conductivity, flexibility, breathability, and waterproofing capabilities.

**Figure 8:**
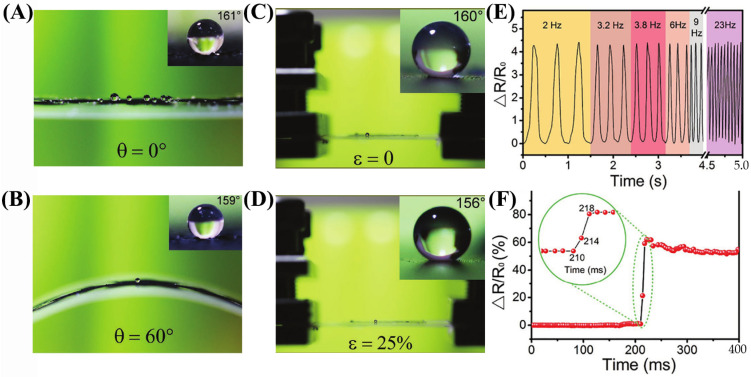
(**A**,**B**) Bending 0° and 60° contact angle droplet size. (**C**,**D**) Droplet contact angle magnitudes at 0% and 25% stretching. (**E**) Resistance change at 0~50% strain at different frequencies. (**F**) The 0% to 5% strain and transition strain sensor response time.

Zhang et al. [92] developed a biomimetic patch that enhances the adaptability and comfort for human skin through its breathability and multi-mechanism adhesion properties. The patch incorporates conical through-holes and hexagonal microgrooves to facilitate the directional transport of sweat from the skin to the air, resulting in improved air permeability. Additionally, the patch exhibits a friction force of 2.26 N under dry conditions and 1.86 N under wet conditions. The biomimetic patch demonstrates exceptional sensing performance within the required 1 kHz bandwidth range for ECG signal detection. Moreover, the patch maintains signal stability even after repeated use, with a gradual decrease in signal intensity observed after 100 cycles. The patch offers a comfortable wearing experience, allowing continuous usage for over 12 h without causing any skin discomfort to the wearer. Li et al. [93] prepared a smart coating with high flexibility and versatility through spraying and ethanol treatment of multi-walled carbon nanotubes dispersed in thermoplastic solution. The coating not only imparts a superhydrophobic surface to the substrate material in various forms (Figure 8A–D) but also is sensitive to stretching, bending, and torsion. The coating has excellent sensitivity (measurement factor of 5.4–80) (Figure 8E), high resolution (bending~1 degree), fast response time (<8 ms) (Figure 8F), stable response over 5000 stretch-relax cycles, and wide sensing range (stretch: more than 76%, bending: 0–140°, torsion: 0–350 rad m^−1^).

### 5.2. Motion Detection Function of Flexible Sensors

With the ongoing advancements in wearable electronic products, flexible robots, and exoskeletons, there is an increasing demand for sensors capable of accurately modeling and monitoring human motion to facilitate human–computer interaction. Traditional rigid motion sensors pose challenges in integrating with the human body and impose motion constraints. With the continuous development of flexible materials and the emergence of new stretchable conductors, flexible sensors have emerged as the preferred solution to address the aforementioned challenges. Flexible sensors offer numerous advantages such as biocompatibility, deformability, and wearability. They not only address the limitations of rigid sensors in terms of poor human–machine integration but also overcome the constraints imposed by experimental environments, enabling motion measurement in diverse real-life scenarios [94]. Flexible sensors have the capability to detect subtle motion signals in various body parts, including the knee, abdomen, arm, and face. Furthermore, they can differentiate between signals generated by different vocal modes when saying different words.

**Figure 9:**
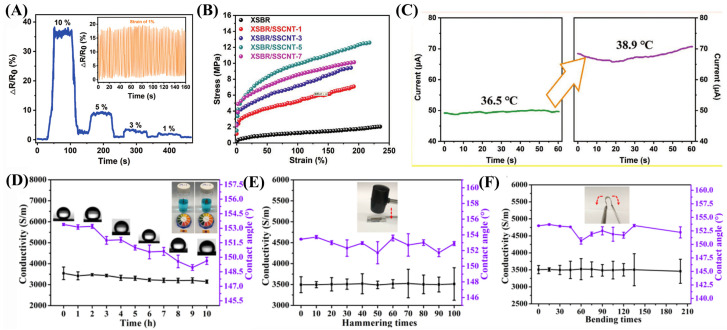
(**A**) Relative resistance variation under reduced gradually step strain from 10% to 1% applied strain. The inset drawing displays the durability of XSBR/SSCNT-5 under 1% strain. (**B**) The tensile stress-train curves of the neat XSBR and XSBR/SSCNT sensors with varying CNTs content. (**C**) Current changes during running and brings down an “artificial fever”. (**D**) CA and conductivity changes in the process of acid exposure. (**E**,**F**) Changes in CA and conductivity for longitudinal hammer and bending of more than 135°.

Sahoo et al. [95] prepared a stretchable, superhydrophobic, and transparent polydimethylsiloxane/carbon nanotube strain sensor by directly spraying CNT solution onto PDMS nano-corrugated substrates. The coating not only offers a superhydrophobic surface but also exhibits responsiveness to stretching, bending, and torsion, which is beneficial to the application of flexible sensors. The sensor maintains a strong response even after 5000 stretch–relax cycles and 10,000 cycles of torsion ranging from 0° to 20°. It senses strain up to 80% in stretching, bending up to 140°, and rotation up to 90°. Lin et al. [96] used carboxyl styrene butadiene rubber (XSBR) and hydrophilic sericin (SS) noncovalent modification of carbon nanotubes (CNTs) as the foundation to design a hydrogen bond crosslinking network for the preparation of multifunctional sensor. The sensor can detect weak deformation and large deformation at the same time and has high elongation (>217%), a low detection limit (<1%) (Figure 9A), extra strength (>12.58 MPa) (Figure 9B), high sensitivity (>25.98), and a low permeability threshold (<0.504 wt%). In addition, the preparation of a sensor with good heat sensitivity (0.01636 °C−1) and high resolution (2.4 °C) (Figure 9C) in the application of body temperature measurement is realized. It has good conductivity and stability (Figure 9D–F).

### 5.3. Flexible Sensor Combined with Anti-Electromagnetic Interference

Capacitive sensors are commonly used in flexible sensors due to their low energy consumption, fast response, and ease of fabrication. However, the practical application of flexible capacitive sensors is limited to laboratories with strict environmental control due to the influence of motion, nearby objects, and low levels of electromagnetic interference. At present, many researchers are developing sensors with anti-electromagnetic interference functions.

**Figure 10:**
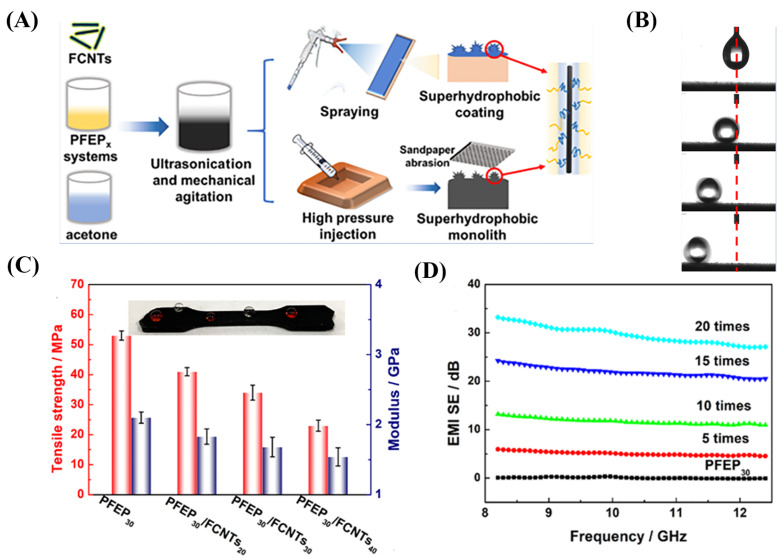
(**A**) Flexible sensor overall and superhydrophobic coating manufacturing process diagram. (**B**) Water droplets on PFEP_30_/FCNT_40_ dripped with rolling consecutive video frames. (**C**) Mechanical property changes of the tension sensor. (**D**) Electromagnetic interference resistance on the surface of the spraying at different times.

Luo et al. [97] investigated polypropylene (PP) fabric conductive silver nanoparticles using oxygen plasma treatment and Fe_3_O_4_ nanoparticles/PDMS hybrid coating. These were prepared and had an outstanding electro-photo-thermal effect and electromagnetic interference shielding performance, with a better superhydrophobic response than that of fabric composite material. The prepared fabric is as high as 108.8 S/cm conductive composite materials, can reach 56.1 dB x-band EMI shielding effectiveness (SE), and has more than a 30% incident electromagnetic wave. The fabric composite material prepared has a superhydrophobic PDMS coating and self-cleaning properties. Ma et al. [98] proposed a more efficient approach to preparing superhydrophobic nanocomposites with fluoride characteristics (Figure 10A), which can be applied as a whole or as a coating on various substrates. In this experiment, long-chain fluorinated epoxy resin (PFEP) with excellent water repellency (Figure 10B) and oil resistance was utilized. Four-fluorine phenyl epoxy resin (FEP) with PFEP has good thermodynamic compatibility and improves the mechanical properties of the matrix (Figure 10C). Additionally, grafted perfluorinated and flexible spacer carbon nanotubes (FCNTs) were incorporated to ensure a good interface roughness. The synthesized PFEP_30_/FCNT_40_ exhibited superhydrophobic and oleophobic properties, excellent thermal conductivity (1.33 W·m^−1^·K^−1^), electronic conductivity (232 S m^−1^), and an electromagnetic interference shielding performance (Figure 10D) (8.2~12.4 GHz, 200 μm) of approximately 30 dB.

### 5.4. Superhydrophobic Surface and Anti-Icing/De-Icing Union

Flexible sensors can perform superhydrophobic surface processing and can be used in vehicles, camera equipment [99], and aircraft window surfaces to prevent frost, freezing, and defrosting. The superhydrophobic flexible sensor itself, with its superhydrophobic function, can not only slow down freezing and prevent ice formation, but it can also detect the thickness of snow or ice covering the surface through the sensor to adjust the deicing power or trigger an alarm [25]. Heating, defrosting, and deicing methods have active, passive, and human management strategies. However, these methods can be expensive and may have artificial limitations. Furthermore, the anti-icing and deicing of a surface can be achieved using electric heating methods with superhydrophobic flexible sensors, which are convenient and cost-effective.

**Figure 11:**
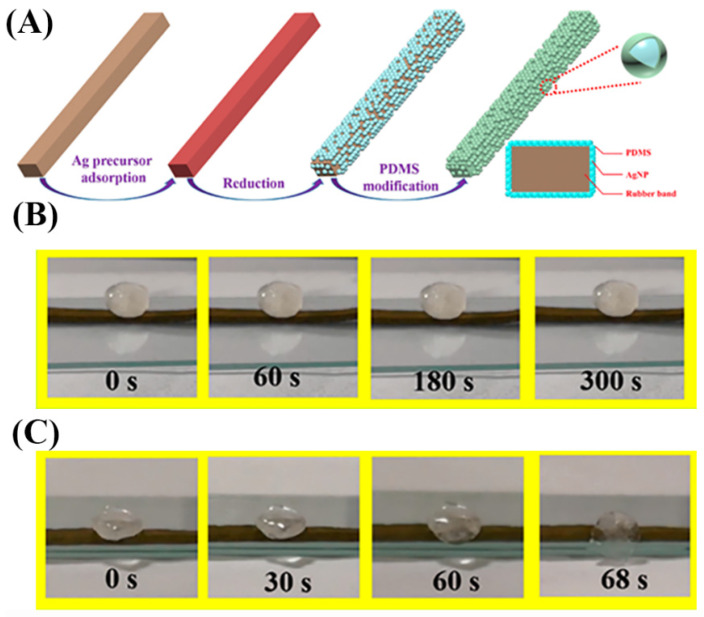
(**A**) Schematic showing the superhydrophobic composite materials’ preparation. (**B**,**C**) Deicing performance of RB/AgNPs/PDMS material with no applied voltage and add 4 V voltage.

Wang et al. [100] employed silver nanoparticles (AgNPs) as a surface decoration on rubber bands (RB) to form a conductive shell (Figure 11A). Subsequently, the RB/AgNPs were modified with polydimethylsiloxane (PDMS) to achieve superhydrophobicity, enhancing corrosion resistance and interfacial adhesion between the AgNPs. This composite material has good superhydrophobic (contact angle is 156°) and self-cleaning performance. Furthermore, the material maintained its hydrophobicity even after undergoing tensile release cycles. In addition, the prepared RB composite material has good tensile properties and high sensitivity, the elongation at break is more than 900%, the strain response is 60%, and the strength can reach 3.6 × 10^8^. Figure 11 B,C shows the superhydrophobic materials’ preparation and electro-thermal deicing capability. With a 4 V voltage, the saturation temperature of the composite material can reach 53 °C. Wang et al. [101] introduced the incorporation of natural fish antifreeze proteins (AFPs) into a chemically crosslinked polyacrylamide/methyl acrylic acid sodium (P (AAm/MAANa)) hydrogel system and a physically crosslinked poly (vinyl alcohol) hydrogel system. These systems exhibit excellent electrical conductivity, shape adaptability, frost resistance, and biocompatibility. The ice crystal inhibition ability of AFPs surpasses that of traditional antifreeze solutions, enhancing the low-temperature performance of the hydrogel. The hydrogel sensor can withstand tensile stress up to 700 kPa and strain within the range of 600% to 1000%. The strain sensor sensitivity (GF) is 12.9, with a response time of 0.17 s. At a strain of 50%, the sensor demonstrates good stability with over 500 cycles of sensing capability.

## 6. Future Perspectives

This article summarizes the construction mechanisms and practical applications of superhydrophobic flexible sensors in human health detection, motion detection, anti-electromagnetic interference, and traffic direction. While the characteristics of superhydrophobic flexible sensors broaden the application range of traditional sensors, there are still several issues associated with superhydrophobic flexible strain sensors:

(1) The hydrophobic and rough surface structure of the flexible sensor changes after stretching or bending, resulting in weakened roughness and hydrophobicity. Therefore, improving the mechanical properties of the sensor is necessary to enhance superhydrophobic stability.
(2) The drugs used in the preparation of superhydrophobic flexible sensors sometimes contain toxic chemicals, which are harmful to experimenters and contribute to environmental pollution. Finding suitable sensing and superhydrophobic materials is crucial to reduce preparation costs and promote green manufacturing.
(3) The superhydrophobic flexible strain sensor has expanded the range of multifunctional applications compared to its ordinary counterpart. However, further research and improvement are needed in areas such as skin affinity, air permeability, and comfort.
(4) Most superhydrophobic flexible strain sensors face challenges due to their complex and time-consuming preparation processes. Therefore, it is crucial to develop preparation methods that are efficient, cost-effective, and suitable for commercial production.

## Data Availability

Not applicable.
